# A Personalized Electronic Movie Recommendation System Based on Support Vector Machine and Improved Particle Swarm Optimization

**DOI:** 10.1371/journal.pone.0165868

**Published:** 2016-11-29

**Authors:** Xibin Wang, Fengji Luo, Ying Qian, Gianluca Ranzi

**Affiliations:** 1 Chongqing University of Posts and Telecommunications, School of Software Engineering, Chongqing, China; 2 Chongqing Engineering Research Center of Software Quality Assurance, Testing and Assessment, Chongqing, China; 3 School of Civil Engineering, The University of Sydney, NSW, Sydney, Australia; 4 State Key Laboratory of Power Transmission Equipment & System Security and New Technology, Chongqing University, Chongqing, China; Beihang University, CHINA

## Abstract

With the rapid development of ICT and Web technologies, a large an amount of information is becoming available and this is producing, in some instances, a condition of information overload. Under these conditions, it is difficult for a person to locate and access useful information for making decisions. To address this problem, there are information filtering systems, such as the personalized recommendation system (PRS) considered in this paper, that assist a person in identifying possible products or services of interest based on his/her preferences. Among available approaches, collaborative Filtering (CF) is one of the most widely used recommendation techniques. However, CF has some limitations, e.g., the relatively simple similarity calculation, cold start problem, etc. In this context, this paper presents a new regression model based on the support vector machine (SVM) classification and an improved PSO (IPSO) for the development of an electronic movie PRS. In its implementation, a SVM classification model is first established to obtain a preliminary movie recommendation list based on which a SVM regression model is applied to predict movies’ ratings. The proposed PRS not only considers the movie’s content information but also integrates the users’ demographic and behavioral information to better capture the users’ interests and preferences. The efficiency of the proposed method is verified by a series of experiments based on the MovieLens benchmark data set.

## Introduction

With the growth in computer networks, information technology and availability of online resources, electronic commerce (E-commerce) has grown extensively over the last decades. Nowadays, the large amount of information available to users is not always assisting them in making decisions because useful and relevant information it is readily distinguishable, i.e. information overloading. To partly address this problem, personalized recommendation systems (PRS) have been developed within the discipline of service computing to assist users in identifying possible products or services of interest based on their preferences. This is usually achieved by extrapolating from historical data of users’ preferences and online behaviors possible recommendations of services and products that might be relevant and of interest to the users.

The underlying techniques used in most of the state-or-the-art recommendation systems can be generally classified into two classes: content-based recommendation (CBR) techniques and collaborative filtering (CF) techniques. In particular, CBR selects items suitable for a user by comparing the representations of the content and user interest model [1], while CF utilizes explicit or implicit ratings from many users to recommend items to a user [2]. Content-based methods are limited in their applicability because based on the textual information of items only. Typically, a profile is formed for an individual user by analyzing the content of items in which s/he is interested (e.g., movie name, director, description, etc.) and additional items can be then inferred from this profile. CF algorithms are widely applied in areas in which the product contents are non-textual, such as music recommendation [3], news recommendation [4], movie recommendation [5], and product recommendation [6].

CF based recommendations can be further subdivided into memory-based and model-based algorithms. The memory-based CF algorithms find neighbors for an active user (new user) and rely on the neighbors’ preferences to predict the preferences of the active user [7]. Shortcomings of the memory-based CF algorithms include the over simplicity of the similarity calculation and the high computational complexity. The implementation of model-based CF algorithms starts from the development of a model from the historical data that is then used to predict new preferences for an active user [8]. Currently, many machine learning methods rely on the model-based CF, such as the Backward propagation (BP) neural network [9], Adaptive learning [10], Linear Classifier [11], Bayesian learning [12], Gradient boosting [13], and Graphic neural network [14].

Compared with many other machine learning approaches [15, 16, 17, 18], the support vector machine (SVM) approach has many advantages. For example, a solution identified with the SVM has the characteristic of an overall optimum and a strong generalization ability [19]. It is worth highlighting that choices of parameters of a SVM heavily influence its prediction accuracy [20]. To date, many heuristic techniques such as grid search (GS), genetic algorithms (GA), and particle swarm optimization (PSO) have been used for the parameter optimization of SVM [21, 22]. Compared with other methods, PSO possesses excellent global search capability and can be easily implemented [23]. Despite this, the standard PSO has some drawbacks such as relapsing into local optimum, slow convergence speed, and low convergence precision in the later evolution.

This paper presents a new personalized recommendation system for electronic movies whose particularities (and contributions) rely on the developments of an improved PSO (referred to in the following as IPSO) and of a support vector machine (SVM) based regression model. In the proposed IPSO, the evolution speed factor and aggregation degree factor of the swarm are introduced to improve the convergence speed, and the position-extreme strategy is used to avoid the search process plunging into the local optimum. In each generation, the inertia weight is updated dynamically based on the current evolution speed factor and aggregation degree factor, which makes the algorithm attain effective dynamic adaptability. The proposed IPSO has stronger global searching performance than the standard PSO, and can yield more accurate prediction results in the proposed recommender system. With the use of the SVM based regression model, the proposed recommendation method overcomes the limitations of the traditional CF methods. Compared with traditional CF methods that only use historical rating data to calculate similarity, the proposed PRS not only utilizes the user’s demographic information but also relies on their rating information. In the implementation of the proposed PRS, the movie data is firstly classified and then the ratings of the testing movie data are predicted. This procedure limits the movie data in the same category range, reduces the forecasting range, and thus enhances the forecast accuracy.

## Background

### 2.1 Basic Principles of SVM

Denoting the training data set as {(*x*_1_,*y*_1_),⋯,(*x*_*l*_,*y*_*l*_)} ∈ *R*^*n*^ × *R*, where *x*_*i*_ is the input vector; *y*_*i*_ is the output value; and *l* is the total number of the training data. Then the relation between *x*_*i*_ and *f*(*x*_*i*_) can be defined as a regression model:

$$y_{i}=f\left(x_{i}\right)=\left(\omega \cdot x_{i}\right)+b$$ (1)

where *ω* is the inertia weight vector; *b* is the pre-specified threshold. *ω* and *b* are determined by following linear optimization model:

$$\underset{\omega ,b,\xi^{\left(*\right)}}{\min}\;\frac{1}{2}∥\omega {∥}^{2}+C\sum_{i=1}^{l}\left(\xi_{i}+\xi_{i}^{*}\right)$$ (2)

$$s.t\;.\;\left\{ \begin{array}{l} \left(\left(\omega \cdot x_{i}\right)+b\right)-y_{i}\leq \varepsilon +\xi_{i},i=1,\cdots ,l\\ y_{i}-\left(\left(\omega \cdot x_{i}\right)+b\right)\leq \varepsilon +\xi_{i}^{*},i=1,\cdots ,l\\ \xi_{i}^{\left(*\right)}\geq 0,i=1,\cdots ,l \end{array}\right.$$ (3)

where *ξ*^(*)^ is slack variable; *C* is punishment coefficient; and *03B5* is insensitive loss function. *ξ*^(*)^ guarantees the satisfaction of constraint condition; *C* controls the equilibrium between the model complexity and training error; *ε* is a preset constant which controls the tube size.

Assuming a transform *ϕ*: *R*^*n*^ → H, *x* ↦ *ϕ*(*x*) which makes *K*(*x*,*x*') = *ϕ*(*x*)⋅*ϕ*(*x*'), where (⋅) denotes the inner product operation. If a kernel function *K*(*x*,*x*') satisfies the Mercer condition, according to the functional theory, it corresponds to the inner product of a transform space. The nonlinear regression model can thus be estimated as:

$$y=f\left(x\right)=\sum_{i=1}^{l}\left({\overset{-}{\alpha }}_{i}^{*}-{\overset{-}{\alpha }}_{i}\right)K\left(x_{i},x\right)+\overset{-}{b}$$ (4)

$$s.t.\;\left\{ \begin{array}{l} \sum_{i=1}^{l}\left({\overset{-}{\alpha }}_{i}^{*}-{\overset{-}{\alpha }}_{i}\right)=0\\ 0\leq \alpha_{i}^{\left(*\right)}\leq C,i=1,\cdots ,l \end{array}\right.$$ (5)

where ${\overset{-}{\alpha }}^{\left(*\right)}={\left({\overset{-}{\alpha }}_{1},{\overset{-}{\alpha }}_{1}^{*},\cdots ,{\overset{-}{\alpha }}_{l},{\overset{-}{\alpha }}_{l}^{*}\right)}^{T}$ is the solution.

In this study, we use the Gaussian function as the kernel function in the form of *K*(*x*,*x*') = exp(−‖*x*−*x*'‖^2^ / *σ*^2^), where *σ* (can also be expressed as g) is the kernel parameter. *σ* precisely defines the structure of high dimensional feature space, and thereby controls the complex nature of the final solution. The selection of parameters *C* and *σ* is critical to the performance of SVM and consequently impacts the generalization and regression efficiency

### 2.2 Standard PSO Algorithm

PSO is a heuristic based optimization algorithm proposed by Kennedy and Eberhart in 1995 [24], and has been applied in many applications [25, 26, 27, 28]. Denoting a swarm consisting of *n* particles; each particle has a position vector *X*_*i*_ = (*x*_*i*1_,*x*_*i*2_,⋯,*x*_*iD*_) and a velocity vector *V*_*i*_ = (*v*_*i*1_,*v*_*i*2_,⋯,*v*_*iD*_), where *i* = 1,2,⋯,*n*. Each particle represents a potential solution to the given optimization problem in a *D*-dimensional search space. In each generation, each particle is accelerated toward its previously visited best position and the global best position of the swarm. The best previously visited position of the *i*-th particle is denoted as *P*_*i*_ = (*p*_*i*1_,*p*_*i*2_,⋯,*p*_*iD*_); the best previously visited position of the swarm denotes *P*_*g*_ = (*p*_*g*1_,*p*_*g*2_,⋯,*p*_*gD*_). The new velocity value is then used to calculate the next position of the particle in the search space. This process repeats until the pre-set termination criterion is achieved. The update of velocity and position vectors of a particle can be mathematically formulated as:

$$v_{id}^{l+1}=w\times v_{id}^{l}+c_{1}\times rd_{1}^{l}\times \left(p_{id}^{l}-x_{id}^{l}\right)+c_{2}\times rd_{2}^{l}\times \left(p_{gd}^{l}-x_{id}^{l}\right)$$ (6)

$$x_{id}^{l+1}=v_{id}^{l+1}+x_{id}^{l}$$ (7)

where *i* = 1,2,⋯,*n*, *d* = 1,2,⋯,*D*; *w* denotes the inertial weight coefficient; *c*_1_ and *c*_2_ are learning factors; $rd_{1}^{l}$ and $rd_{2}^{l}$ are positive random number in the range of [0,1]; *l* is the iteration index; $x_{id}^{l}$ is the position of the particle *i* in the *d*-dimensional space. When applying PSO into SVM, $x_{id}^{l}$ also denotes the current value the parameters *C* and *σ*; *v*_*id*_ ∈ [*v*_max_,*v*_min_] denotes the velocity of a particle *i* in the *d*-dimensional space.

The inertia weight *w* controls the impact of the previous history of velocities on the current velocity. A larger value of *w* facilitates the global exploration, while a small value tends to facilitate the local exploration. In order to balance the global exploration and local exploration capabilities, a linear decreasing inertia weight can be used where *w*(*k*) is reduced linearly in between iterations. This updating process can then be described as:

$$w\left(k\right)=w_{start}-k*\left(w_{start}-w_{end}\right)/T_{\max}$$ (8)

where *k* is the iteration index; *T*_*max*_ is the maximum number of iteration; *w*_*start*_ and *w*_*end*_ are the maximum and minimum values of the inertia weight, respectively.

## IPSO Algorithm

For the standard PSO, when a good solution is found during the early evolution, it is likely that the convergence remains trapped in the local optima. In order to enhance the global searching capability of the standard PSO, the linearly decreasing strategy is designed to self-adaptively adjust the inertia weight *w*. One limitation of this strategy is that with the decrease of *w* in the later evolution, the global searching capability of the algorithm and the diversity of the particles are also weakened. In order to overcome these deficiencies, this paper proposes a non-linearly descending strategy for self-adaptively adjusting the PSO inertia weight.

### 3.1 Evolution Speed and Aggregation Degree Strategy

Let $f\left(P_{g}^{i}\right)$ be the *i*-th generation best global position corresponding to the fitness function value and $f\left(P_{g}^{i-1}\right)$ be the *i*-1-th generation best global position, then we can define the concept of evolution speed as outlined below.

**Definition 1 **Evolution speed *β*:

$$\beta =\frac{\min\left(f\left(P_{g}^{i}\right),f\left(P_{g}^{i-1}\right)\right)}{\max\left(f\left(P_{g}^{i}\right),f\left(P_{g}^{i-1}\right)\right)}$$ (9)

where min(⋅) represents the minimum value function; max(⋅) represents the maximum value function.

**Definition 2 **Aggregation degree *α*:

$$\alpha =\frac{\min\left(f\left(P_{g}^{i}\right),f_{average}\left(t\right)\right)}{\max\left(f\left(P_{g}^{i}\right),f_{average}\left(t\right)\right)}$$ (10)

in which the average fitness function value at the *t*-th generation is determined as:

$$f_{average}\left(t\right)=\frac{1}{N}\sum_{i=1}^{N}f\left(P_{i}^{t}\right)$$ (11)

Based on above definitions, the non-linearly inertia weight can be expressed as follows:

$$w_{\text{nonlinear}}\left(k\right)=w_{start}-A\alpha +B\beta$$ (12)

where *A* is the weight of evolution speed and *B* is the weight of aggregation degree.

### 3.2 Position-Extreme Strategy

To avoid the identification of local optimum, a judgment condition is introduced to influence the selection of the global optimal values in the evolution process. If the global optimal value does not improve in *k* consecutive iterations (that is *k* > *limit*), the algorithm is then assumed to be trapped into a local optimum. In such a case, the search strategy of the particles will change so that the particles escape from the local optimum and start exploring new positions. The corresponding update equations are expressed as follows:

$$x_{id}^{l+1}=rand\left(0,1\right)\cdot x_{id}^{l+1}$$ (13)

$$P_{i}^{l+1}=rand\left(0,1\right)\cdot P_{i}^{l+1}$$ (14)

where *rand*(0,1) represents a random number in the range of [0,1].

### 3.3 Principles of IPSO

Based on the strategies mentioned above, the procedures of the IPSO algorithm can be summarized as follows:

**Step 1: Initialization.** Initialize all particles; initialize parameters of IPSO algorithm including the velocity *V*_*i*_ = (*v*_*i*1_,*v*_*i*2_,⋯,*v*_*id*_) and position *X*_*i*_ = (*x*_*i*1_,*x*_*i*2_,⋯,*x*_*id*_) of each particle. Set the acceleration coefficient *c*_*1*_ and *c*_*2*_, particle dimension, the maximum number of iterations *T*_max_, the maximum number of consecutive times *limit*, the weight of evolution speed *A*, the weight of aggregation degree *B*, the maximum value of inertia weight *w*_*start*_, the minimum value of inertia weight *w*_*end*_, and the fitness threshold *ACC*. $rd_{1}^{l}$ and $rd_{2}^{l}$ are the two random numbers ranging between 0 and 1. *T* is the current number of iterations.
**Step 2: Set values of *P***_***i***_ **and *P***_***g***_**.** Set the current optimal position of the particle *i* as *X*_*i*_ = (*x*_*i*1_,*x*_*i*2_,⋯,*x*_*id*_), i.e. *P*_*i*_ = *X*_*i*_(*i* = 1,2,⋯,*n*), and set the optimal individual in group as the current *P*_*g*_.
**Step 3: Define and evaluate fitness function.** For the classification problems, *Acc* is defined as the classification accuracy:
  $$Acc=\frac{\text{The number of correctly classified samples}}{\text{The total number of samples}}$$ (15)
  For the regression problems, *Acc* is defined as regression error (*MAE*):
  $$Acc=\sqrt{\frac{1}{n}\sum_{i=1}^{n}\left|y_{i}-\hat{y_{i}}\right|}$$ (16)
  where *n* is the number of the samples; *y*_*i*_ is the original values; and $\hat{y_{i}}$ is the forecast value.
**Step 4: Update velocity and position of each particle.** Search for the better kernel parameters according to Eqs (6) and (7). The inertia weight is changed dynamically based on the current evolution speed factor and aggregation degree factor, formulated in Eq (12).
**Step 5: Update the iteration index** by setting *t* = *t*+1.
**Step 6: Check the termination condition.** If *t* > *T*_max_ or Fitness function value < *ACC*, then terminate the algorithm and output the optimal solution; otherwise, go to **step7**.
**Step 7: Judge the global optimum vale unchanged in consecutive *k* times.** If *k* > *limit*, then go to **step 8**; otherwise, go to **step 3**.
**Step 8: Updated the position according to Eqs (13) and (14)**.

## Design of Personalized Movie Recommendation System

This section outlines the design principles of the proposed personalized movie recommendation system.

### 4.1 SVM Classification Based Regression Model

The nonlinear regression problem is solved by using the SVM to establish a classification of the items considered and then to perform the regression based on the obtained classification results. With the proposed IPSO it is possible to optimize the SVM parameters. The detailed steps of SVM classification based regression, that is personalized recommendation for regression method based on SVM classification optimized by IPSO, can be presented as follows:

**Step 1.** Divide the sample data set *S* into *N*_*q*_ (*q* = 1,2,⋯,*s*) classes based on the actual application. where, $S=\overset{k}{\underset{i=1}{\cup }}N_{i},\;N_{n}\cap N_{i}=\emptyset \left(i=1,2,\cdots ,s;\;n\neq i\right)$; *N*_*q*_ is a subset of *S*; ∪ represents the set operator “Union”; ∩ represents the set operator “intersection”; ∅ represents the “Null set”.
**Step 2.** Use a training sample data set of *S* to generate a SVM classifier.
**Step 2.1.** Normalize the sample data.
**Step 2.2.** Select a kernel function and make use of IPSO algorithm to optimize the parameters.
**Step 2.3.** Train the normalized sample data and then obtain the SVM classification model.
**Step 3.** Adopt this classifier to forecast class labels of the testing data. Classify the testing data and get the class label *j* of each sample (*x*_*p*_,*y*_*p*_), where, *p* is the number of the sample.
**Step 4.** For *N*_*q*_ ∈ *type j* ∧ (*x*_*p*_,*y*_*p*_) ∈ *type j*, *M*_*q*_ is a training data set, utilize SVM regression algorithm to predict *y*_*p*_ value of each testing samples.
**Step 4.1.** Normalize *N*_*q*_ and (*x*_*p*_,*y*_*p*_), which belong to the same class *j*.
**Step 4.2.** Select a kernel function and use IPSO algorithm to optimize the parameters.
**Step 4.3.** Train the normalized training data set and establish the SVM regression model.
**Step 4.4.** Adopt the established SVM regression model to forecast *y*_*p*_ value of each testing samples.

### 4.2 Personalized Recommendation Model

The proposed PRS requires the user’s demographic information, user’s behavioral information (“ratings”), and movie’s content information to form a “user-movie” correlation matrix. The correlation matrix is then trained by the training model, after which the movies are ranked. Based on the classification results, the PRS provides a list of recommended movies to the users. Before establishing a classification model, movies are divided into two categories: “like” (recommended) and “dislike” (not recommended), according to the users’ ratings. A movie is rated using the number of the stars to represent the user’s level of preference. The movies with 4 or 5 stars are grouped in the “like” category, and the movies with 1, 2, and 3 stars are included in the “dislike” category.

#### 4.2.1 “User-Movie” correlation feature extraction

**Fig 1:**
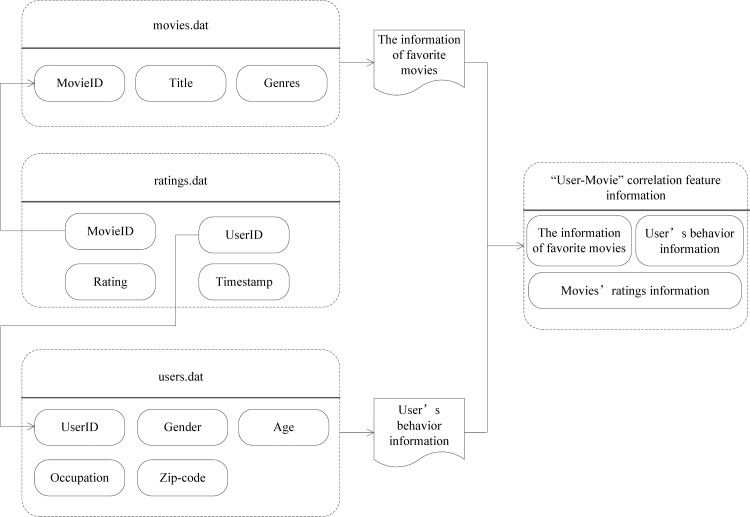
The extraction of relationship feature information of “User-Movie”.

In the proposed movie PRS, the relationship information between the user and movie is essential for establishing the classification model. Based on the realization that the MovieLens data set can be associated by keywords, we use the user’s demographic information, movie’s information, and user’s ratings information about movies to realize the correlation between the user’s preference characteristics and movie’s information. The proposed User-movie correlation feature extraction method is shown in Fig 1.

#### 4.2.2 Personalized recommendation based on SVM

**Fig 2:**
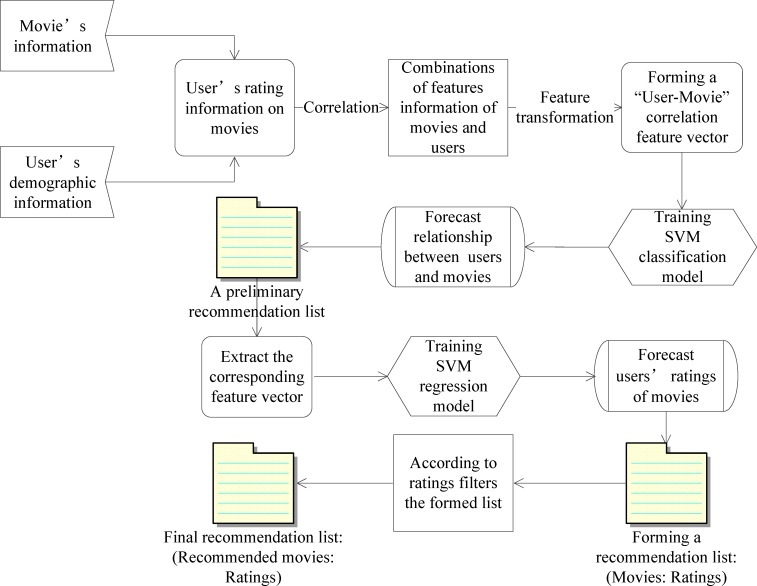
Regression model based on SVM classification for personalized recommendation.

As discussed before, the collaborative filtering methods have some limitations. The UserCF method needs to calculate the similarity between two users based on the items’ rating matrix, while the item-based collaborative filtering (ItemCF) needs to calculate the similarity between two items based on the items’ rating matrix. The computational complexity of the user-based collaborative filtering (UserCF) is related to the number of users, which is proportional to the square of the number of users. For the ItemCF, when the number of the items is large, its computational complexity is also very high, which is proportional to the product of the square of the number of items and the sparsity. Taking into account the user’s demographic information alleviates the “cold start” problem to some extent because such information provides useful hints on the users’ preferences. The personalized movie recommendation model is described in Fig 2.

The classification model based on SVM is built based on the obtained feature vector between users and movies, based on which the movies are classified and a preliminary recommendation list is produced. The latter is then used to build the regression model for the ratings’ forecasts and to form the final recommendation list. In particular, the workflow of the proposed movie recommender system can be summarized in the following steps:

1. The recommender system extracts the movie’s content information and the user’s demographic information, and correlates this information by forming combinations of features information about movies and users;
2. The feature transformation is performed and the “User-Movie” correlation feature vector is formed;
3. The recommender system trains the SVM classification model based on the obtained feature vector, classifies the movies that are without ratings, and forms a preliminary recommendation list according to the classification results;
4. The SVM regression model is trained based on the movies’ feature vector obtained from the preliminary recommendation list;
5. The movies’ ratings are forecasted, therefore narrowing the forecast data range and, as a consequence, improving the forecasting accuracy;
6. The “movie, rating” pairs are obtained based on the preliminary recommendation list and forecasted ratings;
7. The filtering of the list based on the forecasted ratings is carried out, based on which the final recommendation list is established.

## Personalized Recommendation Results and Analysis

### 5.1 Experimental Data Set

To test the performance of the propose recommender system, we select the MovieLens 1M data set as the experimental data set [29]. The MovieLens dataset includes the movie information as well as the users’ demographic information. The MovieLens 1M data set includes 3,900 movie anonymous ratings from 6,040 MovieLens users, which are stored in 3 data files: ratings.dat, users.dat, and movies.dat files. The information of these 3 data files is shown in Table 1.

**Table 1:** Summary of the MovieLens 1M data set.

File name	Description
user.dat	UserID, Gender, Age, Occupation, and Zip-code
	Occupation List: “customer service”, 6: “doctor/health care”, 7: “executive/managerial”, 8: “farmer: UserID, Gender (‘M’ for male and ‘F’ for female), Age (1: Under 18, 18: 18–24, 25: 25–34, 35: 35–44, 45: 45–49, 50: 50–55, 56: 56+), Occupation (0: “other” or not specified, 1: “academic/educator”, 2: “artist”, 3: “clerical/admin”, 4: “college/grad student”, 5: “c”, 9: “homemaker”, 10: “K-12 student”, 11: “lawyer”, 12: “programmer”, 13: “retired”, 14: “sales/marketing”, 15: “scientist”, 16: “self-employed”, 17: “technician/engineer”, 18: “tradesman/craftsman”, 19: “unemployed”, 20: “writer”.)
movie.dat	MovieID, Title, and Genres
	Genres includes: Action, Adventure, Animation, Children’s, Comedy, Crime, Documentary, Drama, Fantasy, Film-Noir, Horror, Musical, Mystery, Romance, Sci-Fi, Thriller, War, and Western.
Ratings.dat	UserID, MovieID, Rating, and Timestamp

### 5.2 “User-Movie” Correlation Feature Extraction

In our movie recommendation system, the relationship information between the user and movie is essential for establishing the prediction model. Based on the MovieLens data set, we use the user’s demographic information, movie’s information, and user’s ratings information about movies to realize the correlation between the user’s preference characteristics and movie’s information. The 3 files store the information of movies, users, and users’ ratings on movies, respectively. The primary and foreign keys of the 3 data tables provide the correlation relationships of the above 3 categories of information. By analyzing the correlation relationships, we extract the users’ behavior and their preference information about the movies, and the “User-Movie” relationship feature vector can be formed.

### 5.3 Classification Results and Analysis

We select 2000 users’ rating data from the MovieLens 1M data set as the experimental data set. For each user, we randomly select 10 data records as testing data, and the remaining data are used as the training set.

For the PSO and IPSO, the parameter settings are as follows: *c*_1_ = 1.5, *c*_2_ = 1.5, *w*_*start*_ = 0.9, *w*_*end*_ = 0.4; the initial speed range of the particles is set to be [−5,5]; the population size is set to be 20; the maximum iteration number is set to be 100. For the SVM prediction model, the parameters of Gaussian kernel are set as follows: *c* ∈ [0,100], *δ* ∈ [2^−10^,2^10^].

**Fig 3:**
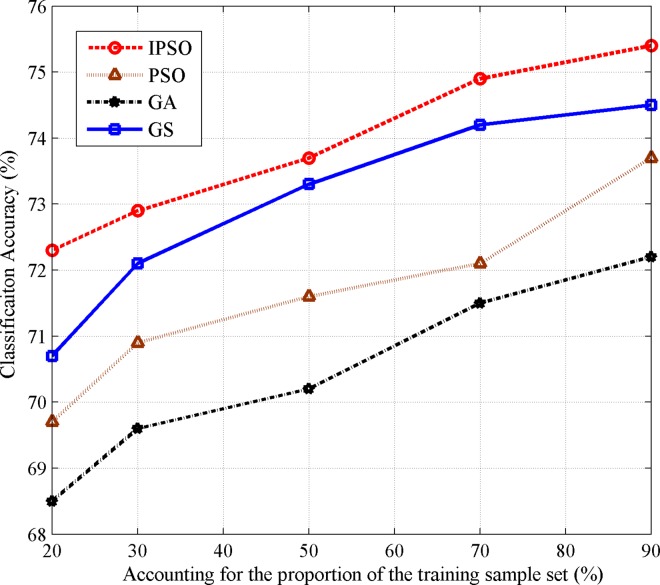
Accuracy of recommended models based on four methods.

In order to prevent errors caused by the random sample selection, we repeat the experiment five times and take the average as the final classification accuracy. Fig 3 reports the comparative results of the IPSO-SVM, PSO-SVM, GA-SVM, and GS-SVM model, respectively. The corresponding optimization results of four kinds of optimization methods.

From Fig 3, it can be clearly seen that IPSO has the best performance on the SVM parameter optimization. When the training samples reache 90% of the entire training set, the classification accuracy of IPSO-SVM reaches 75.4%, which is significantly higher than that of PSO-SVM (73.7%), GA-SVM (72.2%), and GS-SVM (74.5%).

Table 2 shows the average classification accuracy and deviation of IPSO, PSO, GS, and GA after the five experiments.

**Table 2:** The average classification accuracy (%) of four algorithms.

	20%	30%	50%	70%	90%
IPSO	72.3±3.3	72.9±2.8	73.7±2.5	74.9±2.1	75.4±1.9
PSO	69.7±3.6	70.9±3.2	71.6±2.7	72.1±2.4	73.7±2.2
GA	68.5±4.1	69.6±3.9	70.2±3.3	71.5±3.1	72.2±2.5
GS	70.7±5.1	72.1±4.6	73.3±3.8	74.2±3.1	74.5±2.9

From Table 2, it can be seen that in many cases both IPSO and GS reach similar classification accuracies. The deviations of GS are larger than the other three methods, indicating that the GS optimization algorithm is not sufficiently stable for use in practical applications. This is a consequence of the fact that the GS is essentially an exhaustive method whose searching precision is highly related to the step size, and it would be very time-consuming under the smaller step size. This problem does not exist in the proposed IPSO algorithm, and the IPSO can be regarded as a good compromise between classification accuracy and computational time.

### 5.4 Rating Prediction Results and Analysis

**Fig 4:**
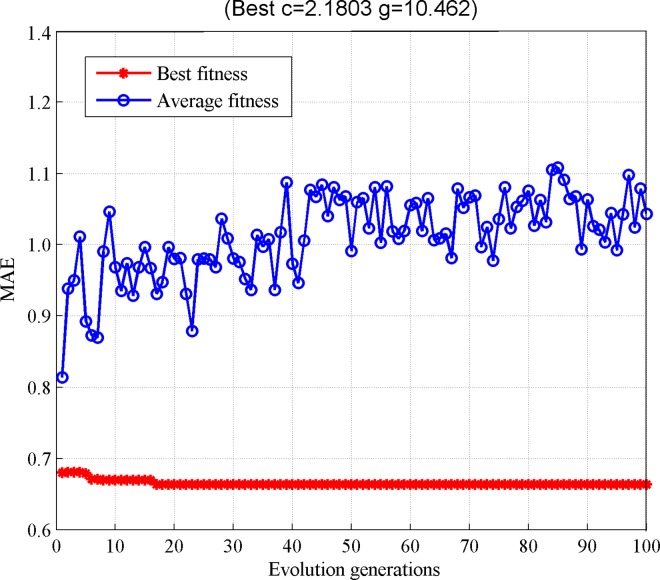
The parameters optimization curve corresponds to IPSO algorithm.

Based on the classification results, the proposed recommender system obtains a preliminary recommendation list. Then, it builds a regression model based on the recommendation list, where the IPSO is utilized to optimize the parameters of SVM. The parameter optimization results are reported in Fig 4. Fig 4 shows that after 100 iterations, IPSO obtains the optimal parameter combination (c = 2.1803, g = 10.462). Fig 4 also shows the profiles of best and average fitness values over the whole population.

The GA and GS are also applied and compared with the IPSO. All three methods adopt the 5-fold cross-validation, and the maximum iteration time is set to be 100. The search range of IPSO is set to be [0, 100]. The settings of GA are basically the same as IPSO. The search step-size of GS is set to be 0.5. The parameters optimization range is set to be [–2^–^^8^, 28].

**Fig 5:**
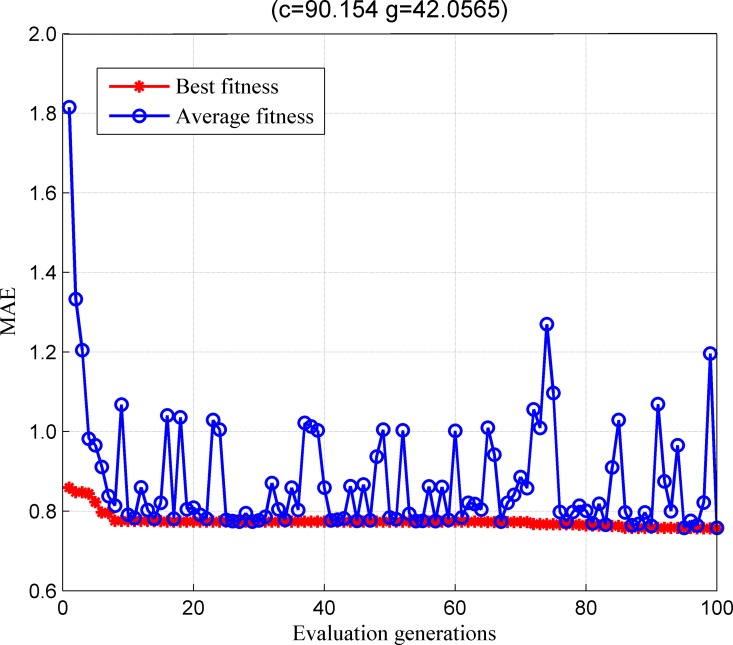
The parameters optimization curve corresponds to GA.

**Fig 6:**
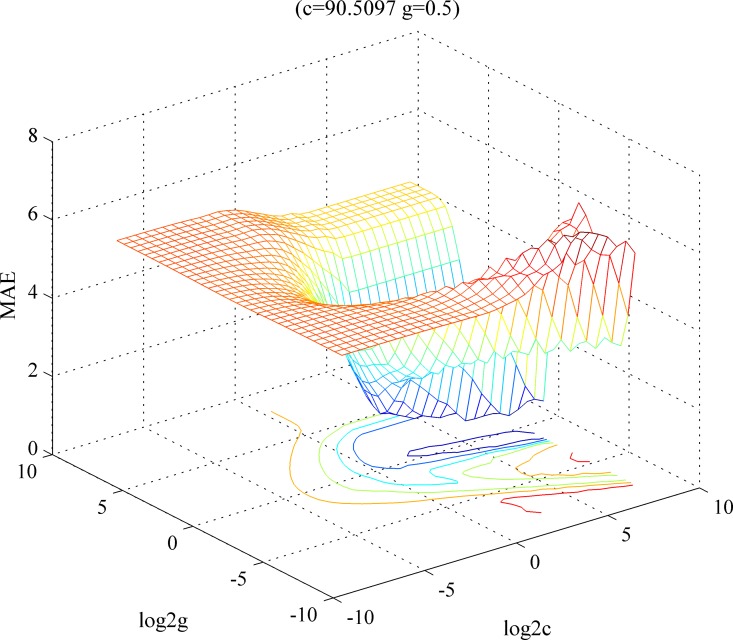
The parameters optimization curve corresponds to GS algorithm.

The optimal parameters optimization results by GA are shown in Fig 5. The results show that after the 100 iterations, GA obtains the optimal parameter combination (c = 90.154, g = 42.0565). The optimal parameters optimization results by GS are shown in Fig 6. After 100 iterations, GS obtains the optimal parameter combination (c = 90.5097, g = 0.5). In the meantime, we observe that the fitness value of particles keep on changing under different parameter combinations. In summary, the results in Figs 4–6 clearly show that in terms of overall fitness, the performance of IPSO algorithm is better than the one of the other two algorithms.

To verify the performance of the proposed regression model on personalized recommendations, several other methods are tested for comparison purposes. These methods include the regression model based on the classification, the item-based collaborative filtering (ItemCF), user-based collaborative filtering (UserCF), (SVM) direct regression model, BP neural network, and multiple linear regression.

**Fig 7:**
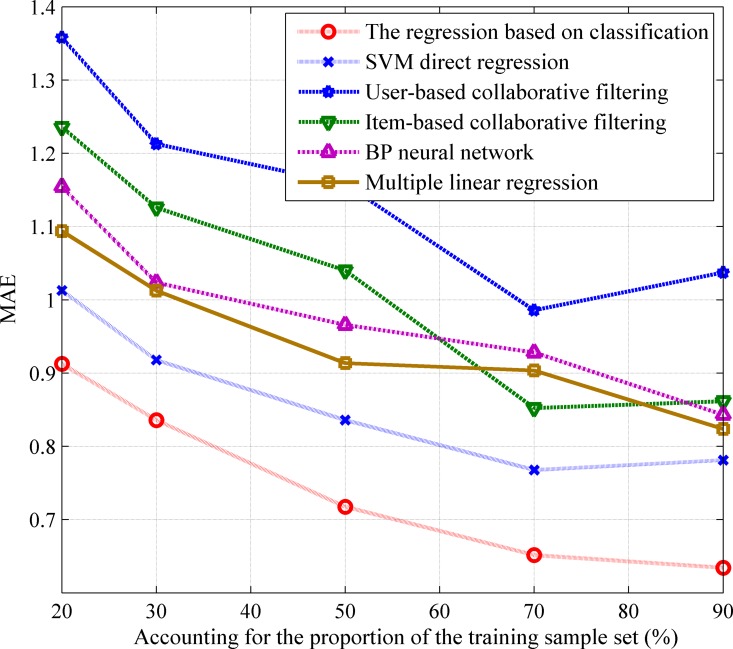
The MAE of ratings based on six methods. It shows the comparison results of the regression based on classification, SVM direct regression, User-based collaborative filtering, Item-based collaborative filtering, BP neural network, and Multiple linear regression.

Fig 7 shows how the actual training sample number accounts for the proportion of the sample set and the corresponding error MAE. The results show that the proposed regression model based classification method has the lowest error, followed by the SVM direct regression method, while the UserCF and ItemCF methods exhibit the highest errors. The errors of BP and Multiple linear regression are very close. These results also show that, with the increase of sample size, the prediction errors also reduce. This is because with the increase of sample size, the numbers of similar users and similar movies also increase, and this helps to enhance the accuracy of the recommendation system.

From the analysis collaborative filtering based recommendation methods, it can be found that the major differences between them and recommendation algorithms based on machine learning methods are as follows: traditional collaborative filtering algorithms only consider the movie’s unilateral rating information, while the machine learning based recommendation algorithms not only use the user’s demographic information and movie’s information, but also use the user’s ratings information about movies. The advantage of this is that it can alleviate the user’s “cold start” problem to some extent. In addition, the user’s demographic information and their rating information can better reflect the user’s preferences.

It is worth mentioning that the proposed PRS firstly builds a SVM classification model to get a movie recommendation list, and then forecast movies’ ratings according to this list. In this way, the range (or number) of the movie samples is narrowed to the movies in the recommendation list, which consequentially enhances the forecast accuracy and efficiency.

## Conclusions

This paper has presented a new rating prediction model for the pre-classification, and later regression, for personalized recommendation. The main advantage of the approach relies on its ability to overcome the limitations of existing collaborative filtering recommendation methods,. In particular, the proposed system starts by establishing a SVM classification model and by identifying a preliminary recommendation list. It then builds a SVM regression model based on the preliminary recommendation list, and predicts items’ ratings. The proposed method is capable of using the items’ content information as well as accounting for the user’s demographic information and behavior information to establish the “user-item” correlation information matrix and to capture the user’s interests and preferences. To improve the performance of the recommendation system, an improved PSO algorithm with the evolution speed factor and the aggregation degree factor (IPSO) is also proposed to optimize the parameters of the model.

To validate the proposed method, experiments are conducted on the public MovieLens data set, and five state-of-the-art recommendation methods are compared. The experimental results show that the proposed model can provide better recommendation results than the other methods.
